# Compound C Inhibits Ovarian Cancer Progression via PI3K-AKT-mTOR-NFκB Pathway

**DOI:** 10.3390/cancers14205099

**Published:** 2022-10-18

**Authors:** Alia Ghoneum, Daniela Gonzalez, Hesham Afify, Junjun Shu, Abigail Hegarty, Jemima Adisa, Michael Kelly, Samuel Lentz, Freddie Salsbury, Neveen Said

**Affiliations:** 1 Departments of Cancer Biology, Wake Forest University School of Medicine, Winston Salem, NC 27157, USA; 2 Department of Obstetrics and Gynecology, Wake Forest University School of Medicine, Winston Salem, NC 27157, USA; 3 Comprehensive Cancer Center, Wake Forest Baptist Health Sciences, Winston Salem, NC 27157, USA; 4 Departments of Urology, Wake Forest University School of Medicine, Winston Salem, NC 27157, USA; 5 Department of Physics, Wake Forest University, Winston Salem, NC 27109, USA

**Keywords:** ovarian cancer, compound C, PI3K, AKT, mTOR, NFκB, platinum-resistance

## Abstract

**Simple Summary:**

Ovarian cancer is a deadly cancer due to its late diagnosis. Despite aggressive surgery and chemotherapy recurrence of a resistant aggressive disease is common. Thus, there is an unmet need to develop new therapeutics that target cancer cells and prevent recurrence and resistance. In the present study, we used multiple approaches to report and validate a novel therapeutic compound, compound C, that targets cancer cells and renders them more sensitive to standard of care therapy. Our study also reports novel mechanism of action of compound C and warrants its further development in the treatment of ovarian cancer patients.

**Abstract:**

Epithelial Ovarian cancer (OvCa) is the leading cause of death from gynecologic malignancies in the United States, with most patients diagnosed at late stages. High-grade serous cancer (HGSC) is the most common and lethal subtype. Despite aggressive surgical debulking and chemotherapy, recurrence of chemo-resistant disease occurs in ~80% of patients. Thus, developing therapeutics that not only targets OvCa cell survival, but also target their interactions within their unique peritoneal tumor microenvironment (TME) is warranted. Herein, we report therapeutic efficacy of compound C (also known as dorsomorphin) with a novel mechanism of action in OvCa. We found that CC not only inhibited OvCa growth and invasiveness, but also blunted their reciprocal crosstalk with macrophages, and mesothelial cells. Mechanistic studies indicated that compound C exerts its effects on OvCa cells through inhibition of PI3K-AKT-NFκB pathways, whereas in macrophages and mesothelial cells, CC inhibited cancer-cell-induced canonical NFκB activation. We further validated the specificity of the PI3K-AKT-NFκB as targets of compound C by overexpression of constitutively active subunits as well as computational modeling. In addition, real-time monitoring of OvCa cellular bioenergetics revealed that compound C inhibits ATP production, mitochondrial respiration, and non-mitochondrial oxygen consumption. Importantly, compound C significantly decreased tumor burden of OvCa xenografts in nude mice and increased their sensitivity to cisplatin-treatment. Moreover, compound C re-sensitized patient-derived resistant cells to cisplatin. Together, our findings highlight compound C as a potent multi-faceted therapeutic in OvCa.

## 1. Introduction

Ovarian cancer (OvCa) is the leading cause of death from gynecologic malignancies in the United States with more than 75% of patients diagnosed at an advanced disease stage [1]. High-grade serous cancer (HGSC) is the most common pathological subtype and accounts for the highest lethality [2]. Recurrence of a chemo-resistant disease is very common due to suboptimal debulking of widespread inaccessible lesions in the peritoneal cavity. Therefore, there is an unmet need to develop new OvCa treatment that not only target tumor cells but also target their interactions within the peritoneal TME that provide a safe haven for resistant and recurrent disease [3]. 

Most patients with HGSC harbor amplification and activating mutations of the catalytic p110α subunit of phosphatidylinositol 3-kinases (PI3K) and is associated with aggressive disease [4,5,6,7,8]. PI3K integrates upstream inputs from growth factors, receptor tyrosine-kinases, and other membrane receptors as integrins, G-protein coupled receptors (GPCRs), cell adhesion molecules, as well as oncogenic signals from oncogenic Ras to promote survival signaling [7,8]. PI3K activates downstream effector, protein kinase B (also known as AKT) [7,8]. AKT activates mammalian target of rapamycin (mTOR) by phosphorylating the Ser2448 which functions through two distinct complexes: mTORC1-Raptor and mTORC2-Rictor [7,8]. When activated, mTORC1 phosphorylates ribosomal S6 kinase-1 (S6K-1) and eukaryote translation initiation factor 4E binding protein-1 (4EBP-1), both are pivotal for cell cycle progression, invasiveness, angiogenesis, and metabolic programming [7,8,9]. While targeting PI3K-AKT-mTOR pathway holds promise as an effective treatment of OvCa, therapeutics targeting them in OvCa and other cancers exhibited limited success. 

Constitutive progressive inflammation is one of the hallmarks of OvCa and is corroborated by the interaction of malignant cells with the cellular components of the peritoneal tumor microenvironment (TME) [10,11,12,13,14,15]. Progressive inflammation activates multiple inflammatory and oncogenic signaling pathways as nuclear factor kappa light chain enhancer of activated B cells (NFκB). The latter is activated/transactivated by multiple oncogenic signaling pathways and subsequently induces the transcription of a myriad of inflammatory mediators that promote OvCa proliferation, invasiveness and chemoresistance [10,11,12,13,14,15,16,17]. Furthermore, p65RelA subunit of NFκB is also activated by PI3K-AKT-mTOR pathway to induce a macrophage phenotypic switch to an inflammatory phenotype [18,19,20]. Moreover, the reciprocal crosstalk between OvCa cells, macrophages, mesothelial cells and other stromal cells results in reciprocal activation of NFκB in these cell types along with the acquisition of inflammatory cancer associated phenotypes further promoting OvCa progression [10,11,12,13,14,18,21,22]. 

Aggressive OvCa cells program their metabolism to meet the increasing demands of rapidly proliferating invasive cells in the unique peritoneal TME [11,23,24,25]. Despite the development of novel chemotherapeutics in the past several decades, the overall and progression free survival of OvCa patients has remained stagnant. Few advances have systematically aimed at targeting the major challenges in OvCa treatment as: the complex OvCa-stromal crosstalk, cisplatin resistance and cellular bioenergetics. Hence, there is a need for therapies that provide more promising outcomes as monotherapies or in combination with the standard of care treatment. 

Dorsomorphin (also known as compound C or BML-275) is a cell permeable ((6-[4-(2-Piperidin-1-ylethoxy) phenyl]-3-pyridin-4-ylpyrazolo [1,5-a] pyrimidine) that was historically used as an inhibitor of adenosine monophosphate kinase (AMPK) to rescue the anti-proliferative actions of AICAR and metformin [26,27]. However, compound C (CC) was found to inhibit other kinases with greater potency than AMPK [28]. Importantly, we found that CC exerts a more potent anti-tumor effect than putative AMPK activators AICAR and phenformin in OvCa cells in vitro (Figure S1). Earlier studies demonstrated that CC exerts an inhibitory role on bone morphogenetic protein (BMP)-SMAD signaling in embryonic development and cancer [29,30]. CC inhibited growth of glioma cells by inducing autophagy [31] and reversed the mesenchymal phenotype of breast cancer cells [27]. In OvCa, CC decreased OvCa cell survival and increased their sensitivity to chemotherapy in vitro and in vivo through the BMP-SMAD pathway [32]. In addition, the anti-angiogenic effect of CC has been reported through an effect on endothelial cells in in vitro assays as well as in murine tumor model of melanoma [33,34]. The anti-angiogenic effect was attributed to its inhibitory effect on BMP receptor, activin receptor-like kinase 1 (BMPR/ALK1) and vascular endothelial growth factor (VEGF) receptors (VEGFRs) in endothelial cells [33,34]. However, the effect of CC on the expression of angiogenic factors including VEGF in tumor cells and other stromal cells in the unique peritoneal TME has not been reported. The anti-tumor effects of CC were reported in the context of OvCa cancer cells; however, its effects on the interactions of OvCa cells within the peritoneal TME, as well as its effect on OvCa bioenergetics have not been elucidated. Herein, we report novel multi-faceted effects of CC as a promising therapeutic for OvCa through inhibition of PI3K-mTOR-NFkB.

## 2. Materials and Methods

### 2.1. Cell Lines

Murine ID8 cell line was earlier described [14,35,36]. Human OvCa cell lines SKOV3, CAOV3, and OVCAR3, as well as human and murine macrophage cell lines U937 and RAW 264.7 were from American Type Culture Collection (ATCC, Manassas, VA, USA). IGROV1 was originally from NCI-60 cell line panel (NCI, Friedrick, MD, USA). Immortalized peritoneal mesothelial cell MESO301 line was a kind gift from Dr. Samuel Mok, PhD (MD Anderson, Houston, TX, USA) and were earlier described [12,13,14,36]. Cell lines were maintained in appropriate cell culture media and were regularly confirmed to be *Mycoplasma*-free at Wake Forest Baptist Medical Center Cell Viral Vector Laboratory (WFBMC-CVVL). Unless otherwise stated, culture media, supplements, antibiotics, and growth factor-reduced matrigel were from Invitrogen (Grand Island, NY, USA), BD Biosciences (Franklin Lakes, NJ, USA),ThermoFisher (Waltham, MA; USA), Sigma-Aldrich (St. Louis, MO, USA), and Gibco (Gaithersburg, MD, USA).

### 2.2. Reagents and Chemicals

Compound C and Cisplatin were purchased from Selleck Chemicals (Houston, TX, USA), and reconstituted in dimethyl sulfoxide (DMSO) according to manufacturer’s recommendations. Lysophosphatidic acid (LPA) was purchased from Sigma.

### 2.3. Antibodies

Monoclonal and polyclonal antibodies against total and phosphorylated PI3K110α, mTOR, phospho-mTOR (Ser2448), total and phospho-AKT (Ser473), total and phospho p65RelA (Ser536) subunit of NFκB, ribosomal protein S6K, phospho-S6K (Thr389), IKKα, HSP90, and horse radish peroxidase (HRP)- and fluorescent-labeled secondary antibodies were purchased from sources described in Table S1.

### 2.4. Primary OvCa Cell Isolation and Maintenance

Freshly excised OvCa tumor tissues and matching ascitic fluids were obtained from Wake Forest Comprehensive Cancer Center under an approved IRB protocol (IRB00052497, PI Said). Briefly, ascitic fluids were centrifuged, and cell pellets were re-suspended in growth media. Tumor tissues were cut into 2–3 mm pieces using a sterile scalpel or scissors and placed on 100 mm dishes pre-coated with matrigel (1:10 in growth media) [37]. Tumor pieces and ascitic fluid cells were cultured in keratinocyte growth media (KGM), and Dulbecco’s Modified Eagle Medium (DMEM) supplemented with 10% fetal bovine serum (FBS), amphotericin B (0.5 µg/mL), penicillin-streptomycin (5000 U/mL) and ciprofloxacin (10 µg/mL). Cells were authenticated by short tandem repeat (STR) analysis at Genetica LabCorp Cell Line authentication services (Burlington, NC, USA).

### 2.5. Proliferation Assays

OvCa cells were seeded in 96 well plates at a concentration of 1 × 10^5^ cells in 100 µL media/well and were allowed to attach for 6 h (considered as 0 h), before treatment with 0–10 µM CC. Proliferation was determined using CYQUANT Cell Proliferation Assay Kit (ThermoFisher) as per manufacturer’s instruction [11,13,14,36,38].

### 2.6. Colony Survival Assay

Cells were seeded in triplicates in six well plates. OVCAR3 and CAOV3 were seeded as 1000 cells/well, SKOV3 at 300 cells/well, and ID8 and IGROV1 at 500 cells/well. Cells were allowed to attach for 24 h, before treatment with 0–10 µM Compound C for 10–14 days. Cells were then washed with PBS and stained with crystal violet solution 0.05% *w*/*v* in 1% formaldehyde, 1% methanol [39]. Colonies > 50 cells were counted using Colony Counter Pen (eCount, Dawsonville, GA, USA). 

### 2.7. Gene Set Enrichment Analysis (GSEA)

Data of gene expression profiling study of SKOV3 treated with 5 µM compound C, *GSE60135* [40] were downloaded from Gene Expression Omnibus (GEO) database. Data were ranked based on fold-change and *p*-values < 0.05. Significant rank-ordered genes were uploaded in the Gene Set Enrichment Analysis (*GSEA*, http://software.broadinstitute.org/gsea/index.jsp, accessed on 3 December 2018) and compared against the Molecular Signatures Database (MSigDB) [41].

### 2.8. Migration, Invasion, and Chemotaxis Assays 

Experiments were carried out in 24-well plates and transwell inserts (8 µm and 3 µm pore-size polycarbonate filters, Corning Costar; ThermoFisher) [11,13,14,36,38]. For invasion assays, filters were coated with 1:5 dilution of reduced growth factor matrigel. Cancer cells (1 × 10^5^ cells in 100 µL serum free media, SFM) were added to the upper chamber of transwells with complete growth media (CGM) in the bottom chamber. For macrophage induced OvCa invasiveness, U937 macrophages (1 × 10^6^ cells in 500 µL CGM) were included in the bottom chamber. For OvCa cell-induced macrophage chemotaxis, OvCa cell lines were grown in monolayers in 12 well plates and U937 cells were added on top of 3µm pore-size polycarbonate filters [11,13,14,36,38]. Migration, invasion, and chemotaxis assays were carried out for 6–8 h, at the end of which the contents of the top chambers were aspirated, and cells were scraped with cotton swabs (ThermoFisher). Cells attached to the bottom surface of the inserts were stained with Diff-Quick (ThermoFisher), counted in five fields *per* insert at 20× magnification [11,12,13,14,38], and imaged with EVOS microscope (ThermoFisher).

### 2.9. Co-Culture Assays

U937 or MESO 301 (1 × 10^6^ cells) were added in the top chamber of 0.4  µm-pore trans-well inserts (Corning, ThermoFisher), with OvCa cells’ monolayers in the bottom chambers for designated times in a 37 °C incubator with 5% CO_2_. Cells from each chamber were collected and used for WB analysis, or real-time quantitative reverse transcription polymerase chain reaction (qRT-PCR) [11,13,14,36,38].

### 2.10. Subcellular Fractionation

Confluent monolayers of SKOV3 and OVCAR3 cell lines were treated with LPA in the presence or absence of compound C for 24 h, after which cells were trypsinized and cell pellets collected. Nuclear and cytoplasmic fractions were prepared by NE-PER™ Nuclear and Cytoplasmic Extraction Reagents (Pierce, ThermoFisher) as *per* manufacturer’s instructions. Protein concentration of each fraction was determined by Bicinchoninic Acid (BCA) assay (Pierce, ThermoFisher) [11,13,14,36,38] and resolved by Western blotting (WB).

### 2.11. Western Blots (WB)

Cells were lysed using radioimmunoprecipitation assay (RIPA) buffer containing 1% NP-40, 0.05% sodium deoxycholate, 0.1% sodium dodecyl sulfate, supplemented with protease and phosphatase inhibitors and protein concentration were determined by BCA [11,13,14,36,38]. Cellular proteins (30 µg) were resolved by 4–20% SDS-PAGE, transferred to polyvinylidene difluoride (PVDF) membranes (Bio-Rad, Hercules, CA, USA), and probed with primary and the appropriate horseradish peroxidase (HRP)-conjugated secondary antibodies (Sigma-Aldrich). Blots were visualized using Amersham Imager (GE Healthcare, Chicago, IL, USA).

### 2.12. Immunofluorescence

OvCa cells were stimulated with 10 μg/mL LPA with and without CC (5 µM) for 18 h in 8-well LabTek chamber glass slides (ThermoFisher) as described previously [13,14]. Cells were fixed with 4% (*w*/*v*) paraformaldehyde for 20 min, washed 3 times using phosphate-buffered saline (PBS), permeabilized with 0.5% (*v*/*v*) Triton X-100 in PBS, and blocked for 20 min at 37 °C with tris-buffered saline with 0.5% bovine serum albumin (BSA), 0.1% glycine, and 0.05% Tween 20 (TBS-BGT). Cells were incubated for 1 h at 37 °C with primary antibody against phosphorylated p65RelA diluted in TBS-BGT followed by washing 3 times with TBS-BGT and incubation with Alexafluor-594 or -488 donkey anti-rabbit secondary antibody for 1 h at room temperature. Slides were mounted in Fluor Gel II (Invitrogen) containing 4′,6-diamidino-2-phenylindole (DAPI). Images were acquired from five fields/well with two replica per experimental condition at 40× magnification using Olympus IX-70 fluorescence microscope (Olympus, Tokyo, Japan). Digital images were analyzed using ImageJ software (64-bit bundled in Java 8 for Windows, NIH, Bethesda, MD, USA).

### 2.13. Plasmids, Lentiviral Packaging, and Viral Transduction

Overexpression plasmids were purchased from Addgene (Cambridge, MA, USA). The following plasmids were used pBabe puro MyrHA-PIK3CA (Addgene# 12523) [42], pBabe-Puro-Myr-Flag-AKT1 (Addgene# 15294) [43], pBabe-Puro-IKBalpha-wt (Plasmid #15290) [43] and control vector pBabe-puro (Addgene# 1764) [44]. Packaging plasmids pMD2.G (Addgene #12259) and pCL-Eco (Addgene #12371) [45]. To generate viral overexpression particles, plasmids were packaged by transfection of human embryonic kidney cells (HEK 293T) using FugeneHD (Promega, Madison, WI, USA) in OPTIMEM (GIBCO) for 6–8 hr, then were replaced by complete growth media. Viral particles in culture media were collected after 48 h, and filter sterilized through 0.4 µm filters, aliquoted and stored in −80 °C [43]. For transduction of OvCa cells, 1–2 mL collected viral particles were added in 7 mL complete growth media to 60–70% confluent monolayers of OvCa cells in presence of 8 µg/mL polybrene for 24 h [43]. Stably transduced cells were selected by 5 µg/mL puromycin in culture media for 72 h and were confirmed by Western blotting and probing with the appropriate antibodies [43].

### 2.14. In Vitro Adhesion Assays

Mesothelial cells (MESO301) were seeded in a Corning Costar 96 Well clear bottom, black assay plates, and were allowed to form a monolayer overnight as previously described [13,14]. OvCa cells (SKOV3, OVCAR3, IGROV1, CAOV3) were fluorescently labelled with CellTracker™ Orange 5-(and-6)-(((4-chloromethyl)benzoyl)amino)tetramethylrhodamine (CMTMR) Dye (Invitrogen) for 30 min at 37 °C, washed, trypsinized and 1 × 10^6^ cells/ well seeded on top of uncoated, and matrigel-coated wells, as well as MESO301 monolayers. OvCa cells were allowed to adhere for 2 h in the presence or absence of 5 µM compound C or DMSO vehicle control. Non-adherent cells were washed, fixed, and visualized using Olympus IX-70 Imager at 594 nm visualized 10× magnification in 10 fields with four replica/experimental condition, and were analyzed using ImageJ analysis software (NIH). 

### 2.15. In Vivo OvCa Cell Homing/Adhesion Assays

Confluent monolayer of ID8 cells were pre-treated with 5 µM of Compound C or DMSO for 18 h and were labelled with CellTracker™ Blue 7-amino-4-chloromethylcoumarin (CMAC) Dye (Invitrogen) for 30 min. C57BL6 mice were injected intaperitoneally with 1 × 10^6^ CMAC-ID8 cells. After four hours, mice were euthanized, omenta were collected in 6 well cell plates and gently washed once with PBS (*n* = 4 mice *per* group). After which, specimens were preserved in 70% ethanol. Fluorescence imaging was immediately conducted using Olympus IX-70 at 460 nm (blue) and visualized in 5 fields/organ at 100× magnification and analyzed using Image J software. 

### 2.16. RNA Extraction and Real-Time Quantitative Reverse Transcription Polymerase Chain Reaction (qRT-PCR)

Total RNA was isolated using RNEasy Kit (Qiagen, Germantown, MD, USA) [11,12,25]. Total RNA (1 µg) was reverse transcribed in a 20µl reaction using iScript cDNA synthesis kit (Bio-Rad). cDNA was amplified using forward and reverse primers (Table S2) with SsoAdvanced Universal SYBR Green Supermix (Bio-Rad). Reaction conditions were as follows: Polymerase activation and DNA denaturation for 30 s at 95 °C, followed by 35 cycles of 95 °C for 15 s and 60 °C for 30 s. PCR was performed in 96-well plates in CFX Connect Real-Time System (Bio-Rad). All experiments were performed in triplicates and were normalized to 18S mRNA as reference housekeeping gene. 

### 2.17. Measurement of Mitochondrial Mass MitoTracker Staining

OvCa cells were seeded in 8-well LabTek slide chambers and were treated overnight with CC (5 µM). Cells were stained with MitoTracker Green (for mitochondrial mass) as previously described [25]. After fixing cells, slides were mounted in fluorogel containing DAPI and covered with coverslips. Images were acquired using Olympus FV1200 Confocal Imager (Tokyo, Japan). Average fluorescence intensity *per* cell was detected by PICO CellReporterXpress image acquisition and analysis software (Molecular Devices, San Jose, CA, USA), and measured by ImageJ Software.

### 2.18. In Vivo Tumor Xenografts

Luciferase-tagged SKOV3 (SKOV3-luc) cells were injected intraperitoneally (2 × 10^6^ cells/100 µL PBS, using 27G syringe needles) in 6–8 weeks old female athymic nude mice (Charles River Laboratories, Wilmington, MA, USA) as earlier described [11,38]. Tumor growth was monitored bioluminescent imaging with IVIS Spectrum In Vivo Imaging System (PerkinElmer, Waltham, MA, USA). One week after tumor cell injection, mice were stratified into four groups: control/PBS, compound C (9 mg/kg/d), cisplatin (1 mg/kg/d), and a combination of CC (9 mg/kg/d) and cisplatin (1 mg/kg/d). Mice received treatment three times/ week for three weeks. Mice were weighed once a week and imaged with IVIS once every three weeks for eight weeks, after which mice were euthanized by isoflurane inhalation and cervical dislocation. Intraperitoneal tumors were dissected, weighed, and measured, and ascitic fluid was collected for further analysis [11,12,13,38].

### 2.19. Immunohistochemistry (IHC)

IHC was performed on formalin-fixed paraffin-embedded sections as earlier described [11,25,38]. After de-paraffinization, antigen-retrieval (by boiling in 0.01% citric acid for 15 min). Sections were incubated with the indicated primary antibodies (Table S1). After washing, sections were developed with secondary antibodies in Vectastain ABC ELITE (Vector Laboratories, Inc., Newark, CA, USA) according to manufacturer’s instructions. Slides were developed by diaminobenzidine (DAB) as a chromogen and hematoxylin as the nuclear counterstain. Negative controls were included omitting the primary antibody. Slides were scanned using Olympus VS120 Automated Slide Scanner (Olympus). Digital image analysis was carried out as earlier described [11,25,38]. The frequency of positive staining was determined by the percentage of positive cells counted in whole tumor section with three tumor sections/experimental condition examined. 

### 2.20. Docking

Coordinates for dorsomorphin were obtained from Pubchem (CID 11524144) [46] and prepared using Babel [47] and AutoDockTools [48] for conversion to a Protein Data Bank (pdb) and Protein Data Bank, Partial Charge (Q), & Atom Type (T) (pdbqt file) respectively. The relevant protein structure files were obtained from the Research Collaboratory for Structural Bioinformatics (RCSB) PDB IDs 1E7V, 4JSV, and 3GUT [49,50,51]. Non-protein atoms were removed manually, and the files prepared as pdbqt files also using AutoDock Tools [52]. The docking was performed using AutoDock Vina [48], using the default exhaustiveness. The 40 Angstrom cubic grid was centered at the relevant middle of the relevant binding pocket for each structure. For p65relA a grid of 80 Angstroms was also tested to see if an alternative binding mode could be found; one was not.

### 2.21. Statistical Analysis

Data were analyzed by two-tailed unpaired Student’s *t*-test, multiple *t*-test, and one- and two-way analysis of variance (ANOVA) with Sidak-Holm test. Differences were deemed significant at *p* < 0.05. GraphPad Prism 7.0 (San Diego, CA, USA).

## 3. Results

### 3.1. Compound C Inhibits OvCa Proliferation and Clonogenic Survival

**Figure 1:**
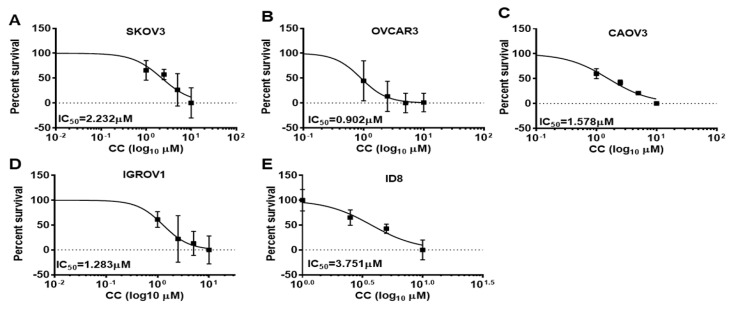
Compound C inhibits OvCa cell proliferation. Compound C decreased the proliferation of (**A**). SKOV3, (**B**). OVCAR3, (**C**). CAOV3, (**D**). IGROV1, and (**E**). ID8 OvCa cell lines. IC_50_ was determined after 48 h time point. Represented is an experiment that was repeated twice (*n* = 4/experimental conditions) with reproducible results.

**Figure 2:**
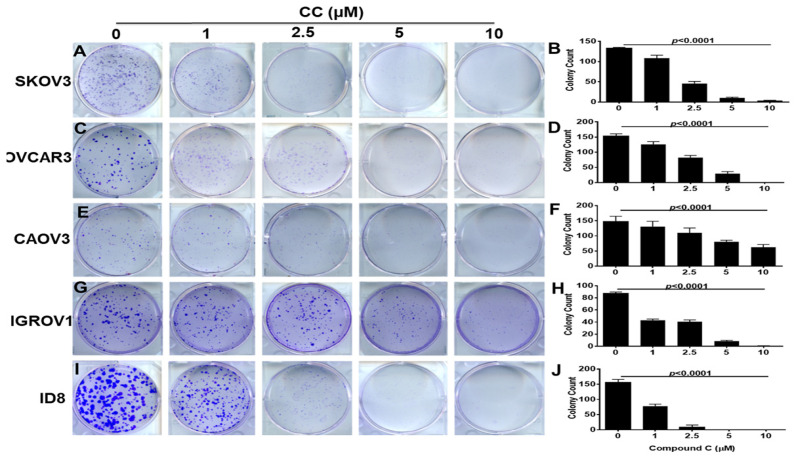
Compound C inhibits colonogenic survival of OvCa cells. Compound C exerted a significant dose-dependent decrease in colony formation of OvCa cell lines: (**A**,**B**). SKOV3, (**C**,**D**). OVCAR3, (**E**,**F**). CAOV3, (**G**,**H**), IGROV1, and (**I**,**J**) ID8. Representative images of the counted colonies. Bars represent mean ± SEM of colonies stained with crystal violet, quantified by colony counter pen. *p*-value using One Way ANOVA. *n* = 3/experimental condition, performed twice.

To determine the effects of CC on OvCa cell malignant phenotype, we treated SKOV3, OVCA3, IGROV1, and CAOV3, and murine ID8 OvCa cell lines with increasing concentrations of CC and determined the effect on proliferation and clonogenic survival. We found that CC exerted an inhibitory effect on OvCa proliferation in the five OvCa cell lines in a time and dose dependent manner (Figure 1A–E). Consistently, CC inhibited clonogenic survival in a dose-dependent manner in the five OvCa cell lines (Figure 2A–J). 

### 3.2. Compound C Inhibits OvCa Cell Migration and Invasiveness

**Figure 3:**
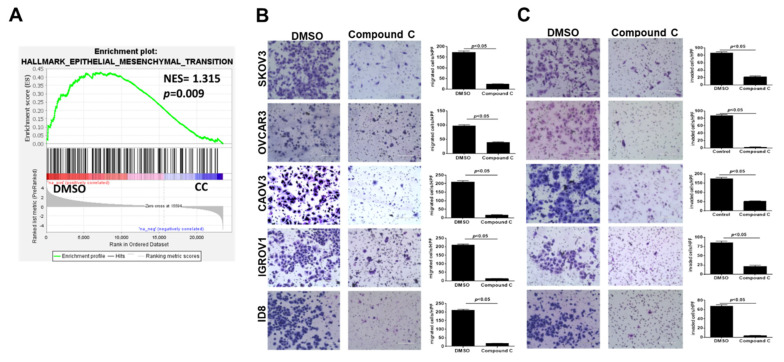
Compound C inhibits OvCa cell migration and matrix invasiveness. (**A**) Gene set enrichment analysis showing inhibition of EMT signature in CC-treated SKOV3 cells. (**B**) CC inhibits OvCa cells migration and (**C**) invasion. Bars represent mean  ±  SEM of migrated and invaded cells counted in 6 high power fields (200× magnifications, *n* = 3/experimental condition, repeated twice). *p*-value using Student’s *t*-test.

Gene set enrichment derived from *GSE60135* of SKOV3 cells treated with CC [40] showed significant inhibition of the epithelial mesenchymal transition (EMT) signature in SKOV3 cells treated with 5 µM of CC (Figure 3A). Thus, we determined the effect of CC on migration and matrix invasiveness of OvCa cells. Consistently, we found that CC significantly inhibited OvCa cell migration and matrix invasion in all OvCa cell lines (Figure 3B,C).

### 3.3. Compound C Inhibits PI3K-AKT-mTOR and NFκB in OvCa

**Figure 4:**
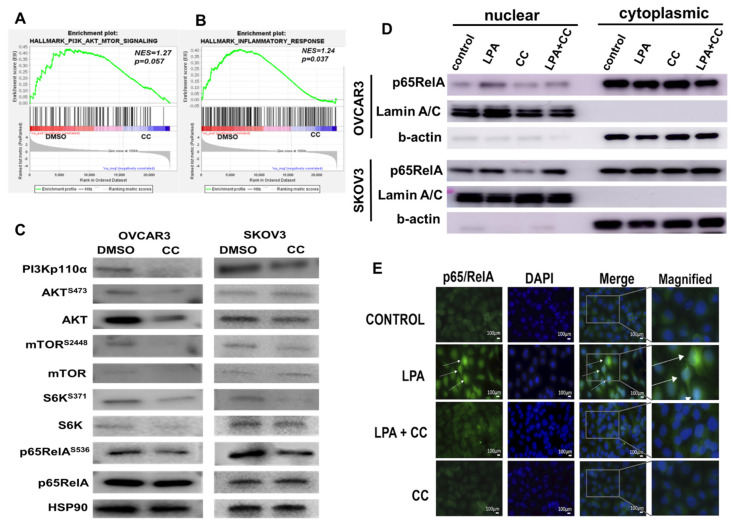
Compound C Inhibits PI3K-AKT-NFκB axis. (**A**,**B**). Gene set enrichment analysis showing inhibition of PI3K pathway and the inflammatory response in CC-treated SKOV3 cells. (**C**). Western blot showing the effect of CC on PI3K-AKT-mTOR-NFκB pathway SKOV3 and OVCAR3 cell lines, with HSP90 as loading control. (**D**). Western blots showing the effect of CC on LPA-induced nuclear localization of p65RelA subunit of NFκB in SKOV3 and OVCAR3 cells. Lamin A/C and b-actin were used as nuclear and cytoplasmic markers respectively. (**E**). Immunofluorescence staining of SKOV3 treated with CC showing inhibition of nuclear translocation of p65RelA in SKOV3 cells by CC (magnification, 400×). White arrows indicate the green fluorescence of nuclear translocation of p56RelA.

Recent reports indicated PI3K-AKT-mTOR and NFκB axis are of the most amplified and hyperactivated pathways in OvCa [8,53,54,55,56]. We have recently shown that the transcripts of the key molecules involved in these pathways are not only associated with poor patients’ survival in TCGA data, but the expression of these transcripts positively correlated with each other as well, suggesting positive feedforward activation [8,53,54,55,56]. In addition, Gene Set Enrichment Analysis (GSEA) of *GSE60135* study revealed inhibition of PI3K-AKT-mTOR as well as inflammatory signatures in CC-treated SKOV3 cells (Figure 4A,B). Given that this axis represents the central hub of oncogenic signaling linking cancer cell proliferation, invasiveness as well as metabolic reprogramming of cancer cells, we sought to determine whether CC exerts its inhibitory effect on OvCa cells through inhibition of this pathway. Compound C decreased the expression as well as the activation and phosphorylation of key regulators in PI3K-AKT-mTOR and NFkB signaling pathways in two OvCa cell lines, SKOV3 and OVCAR3 (Figure 4C and Figure S2). In addition, we found that CC inhibited basal and LPA-induced activation and nuclear translocation of p65RelA subunit of NFκB (Figure 4D,E and Figure S3). 

**Figure 5:**
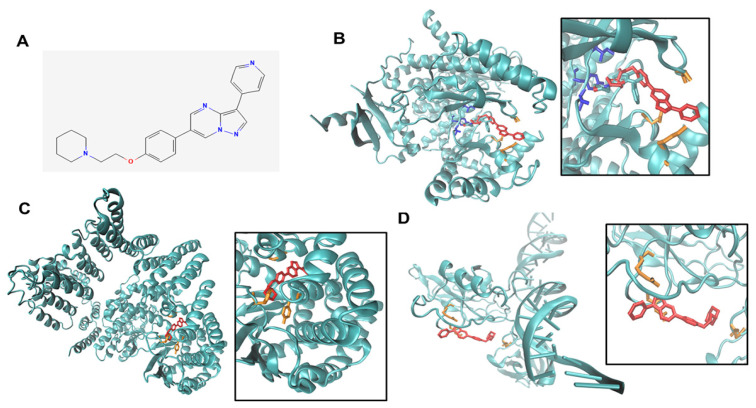
Predicted binding modes of Compound C to PI3K, mTor, p65RelA. (**A**). CC binds in the ligand binding pocket of PI3K, (**B**). CC (red) binds in a similar manner to a PI3K ligand, LY294002, (gray). The key residues for the LY294002-PI3K interactions are VAL 882 TYR 867 ILE 879 (blue) whereas ASP 950, LYS 890, and ALA 805 (orange) are key residues for CC-PI3K interactions. (**C**). CC (red) binds in a pocket in the kinase domain of mTOR. The key residues for mTOR-CC interactions are PRO 1940, PRO 1975, and TYR 2144 (orange). (**D**). CC (red) binds to p65relA is in the DNA binding domain, though weakly. The key residues for p65relA-CC interactions are ASP 80, ARG 84, and ASN 190 (orange).

To confirm the specificity of the inhibitory effect of compound C on PI3K-AKT and NFκB, we used two independent approaches. First, we performed in silico simulation and modeling of the structure of CC and the structures of the key nodes in this pathway. We found that CC (Figure 5A) bound to PI3K-p110α subunit at ASP 950, LYS 890, and ALA 805 (Figure 5B). Computational modeling also revealed that CC binds to mTOR at 130 nM. While the binding is not at the catalytic site, it is nearby in a pocket/cleft in mTOR molecule at residues PRO 1940, PRO 1975, TYR 2144 (Figure 5C). Moreover, in silico modeling also revealed CC binds p65RelA subunit of NFkB near the DNA binding site and three nearest interacting residues, ASP 80, ARG 84 and ASN 190 (Figure 5D). 

**Figure 6:**
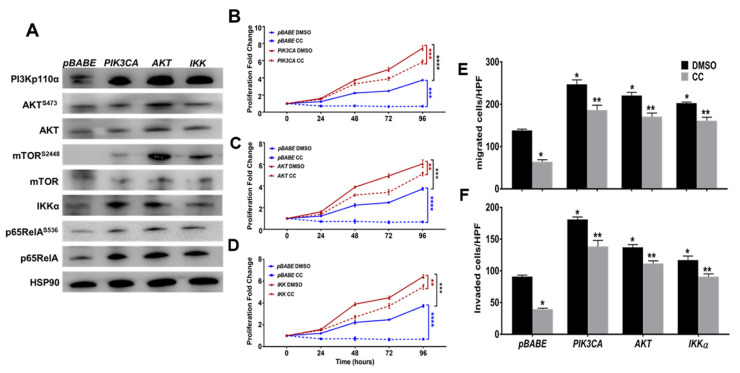
Constitutive activation of the PI3K-AKT-mTOR-NFκB Pathway in SKOV3 mitigated the inhibitory effect of CC. (**A**). Western blots showing overexpression of *PIK3CA*, *AKT* and *IKKA* in SKOV3 cells and *pBABE* as vector control, and HSP90 as loading control. (**B**–**D**). Stable overexpression of *PIK3CA, AKT* and *IKKA* partially rescued the inhibitory effect of CC on SKOV3 proliferation. Line graphs represent the means ± SEM of the fold change in cell proliferation over 96 h. Values were compared to untreated cells 0hr considered as 1; (*n* = 4/experimental condition, repeated twice). * *p* < 0.05, *** *p* < 0.0001, Student’s *t*-test. (**E**,**F**). Constitutive overexpression of *PIK3CA*, *AKT* and *IKK* mitigated the inhibitory effect of CC on migration and invasion. Bars represent the means ± SEM of cell count. Values were compared to untreated cells *pBABE* vector controls (*n* = 3/experimental condition; repeated twice). * *p* < 0.05 compared to *pBABE* vector control treated with DMSO, ** *p* < 0.05 comparing CC-treatment to corresponding DMSO control, Student’s *t*-test. **** *p* < 0.0001.

Secondly, we stably overexpressed constitutively active myristoylated PI3K-p110α catalytic subunit, as well as AKT1 and IKKα, with *pBABE* as a vector control (Figure 6A and Figure S4). Overexpression of myristoylated PI3Kp110α in SKOV3 cells led to modest change in total and phospho-AKT (ser473) but markedly increased phosphorylation of downstream mTOR at ser2448, IKKα as well as total and phosphorylated (ser536) p65RelA subunit of NFκB (Figure 6A and Figure S4). Overexpression of constitutively active AKT1 increased the expression of PI3Kp110α, activation and phosphorylation of mTOR (ser2448), IKKα as well as total and phosphorylated (ser536) p65RelA subunit of NFκB. Consistently, overexpression of IKKα increased the expression of PI3K-p110α, activation and phosphorylation of mTOR (ser2448), as well as total and phosphorylated (ser536) p65RelA subunit of NFκB but had no effect on AKT expression or activation (Figure 6A and Figure S4). These data further confirm the interconnected feedforward activation loop of PI3K-AKT-mTOR-IKKα-NFκB in OvCa cells. Phenotypically, overexpression of myristoylated PI3K-p110α, AKT1, and IKKα significantly increased proliferation of SKOV3 at 72–96 h for PI3K-p110α, and 48–96 h for AKT1 and IKKα, respectively (Figure 6B–D). Importantly, they mitigated the inhibitory effect of CC on cell proliferation (Figure 6B–D). Similarly, overexpression of myristoylated PI3Kp110α, AKT1, and IKKα significantly increased SKOV3 migration and matrix-invasion and mitigated the inhibitory effect of CC (Figure 6E,F). Together, these data further support the specificity of the inhibitory effect of CC on the key nodes PI3K-AKT-NFκB axis. 

### 3.4. Compound C Inhibits OvCa Cells-Mesothelial Interactions In Vitro and In Vivo

**Figure 7:**
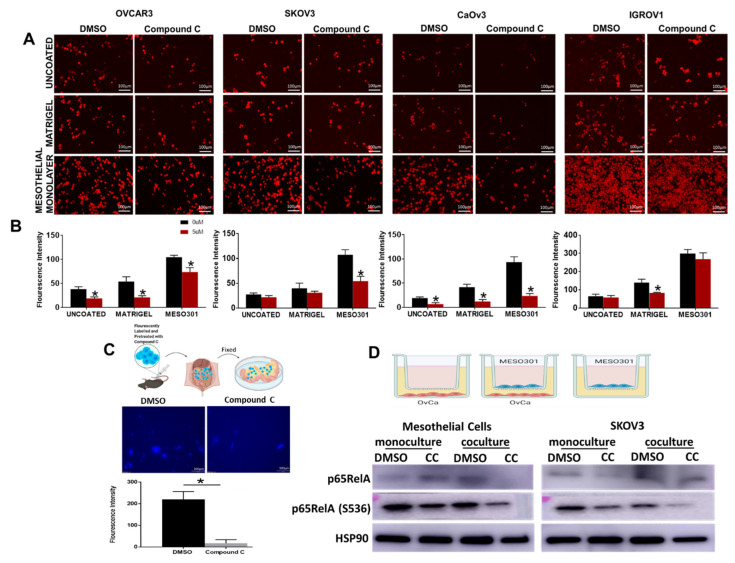
CC Inhibits mesothelial-OvCa crosstalk. (**A**). Representative images of the effect of CC on the adhesion of CMTMR-labelled OvCa cell lines to uncoated, and matrigel-coated plates, or mesothelial cells monolayers (scale bars, 100 µm). (**B**). Bars represent means ± SEM of quantification of fluorescent cells was quantified using Image J. *n* = 6/experimental condition, * *p*  <  0.05, Student’s *t*-test. (**C**). CC reduced adhesion of fluorescent-ID8 cells homing to mesothelium covering the omenta of C57B6 mice. Top, schema of the experiment; middle, photomicrographs of dissected omenta and adherent cells. Bottom, bars representing means ± SEM of fluorescent cells quantified using Image J. *n* = 5/experimental condition, * *p*  <  0.05, Student’s *t*-test. (**D**). Top, schema showing mono and coculture of MESO301 and SKOV3 cells. Bottom, Western blot of the effect of CC on the expression of total and phosphorylated p65RelA in mesothelial and OvCa monocultures and cocultures.

The mesothelial cell monolayer is the first barrier that OvCa cells from the primary tumor encounter for peritoneal colonization and spread [12,13,14,57,58]. To investigate the effect of CC on the ability of OvCa cells to adhere to the mesothelial layer, human OvCa cells were treated with either CC (5 µM) or vehicle (DMSO) for 30 min and were allowed to adhere to uncoated-, and matrigel-coated plates, or mesothelial monolayers on 96 well plates for 2 h [12,13,14]. We found that CC significantly inhibited OvCa cells adhesion to the uncoated-, and matrigel-coated wells as well as mesothelial monolayers (Figure 7A,B). To further verify the effect of CC on OvCa cell chemotaxis and adhesion to the mesothelial surface in vivo, fluorescent-labelled ID8 cells were injected intraperitoneally into C57B6 mice as earlier described [11]. Two hours later, mice were euthanized, omenta and mesentery dissected, fluorescent OvCa cells that homed to and adhered to mesothelial cells covering mesentery and omenta were visualized under a fluorescent microscope, and fluorescent signal quantified. We found that CC significantly inhibited the homing and adhesion of OvCa cells to the mesothelial cells coving the omentum (Figure 7C). To determine the mechanism of the inhibitory effect of CC on OvCa cell-mesothelial cell interactions, we treated OvCa cells and mesothelial cells in mono and co-cultures with CC and determined the effect on both cell types. Consistent with our earlier reports [10], co-cultures of OvCa cells with mesothelial cells increased phosphorylation and activation of p65RelA subunit of NFkB. Compound C decreased phosphorylation and activation of p65RelA subunit of NFκB in mesothelial and OvCa cells in mono and cocultures (Figure 7D and Figure S5) and significantly decreased the transcript levels of target pro-inflammatory markers as IL6, IL8, CCL2, VEGF, IL1β and TNFα in both MESO301 and SKOV3 cells (Figure S6).

### 3.5. Compound C Inhibits OvCa Cell-Macrophage Crosstalk

**Figure 8:**
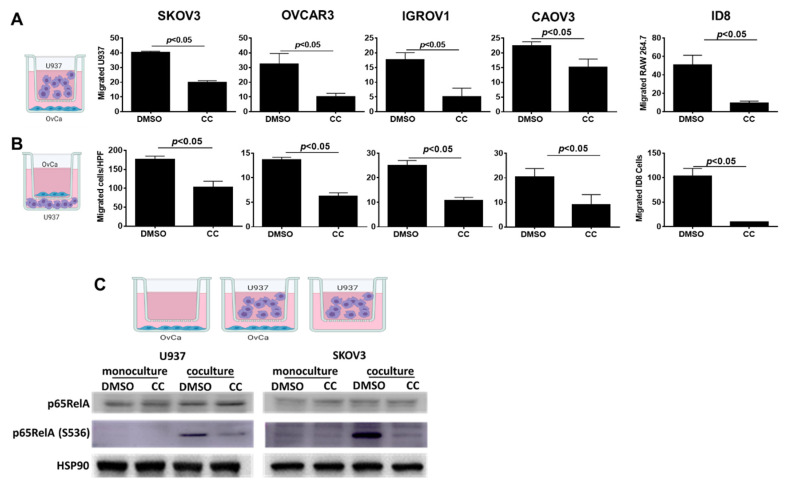
Compound C Inhibited U937-OvCa Crosstalk. (**A**). Schema showing experimental design of macrophage chemotaxis towards OvCa cells, left. The effect of CC on migration of human U937 towards SKOV3, OVCAR3, IGROV1, and CaOV3 cells as well as murine RAW 264.7 towards ID8 cells, right. (**B**). Schema showing experimental design of macrophage induced OvCa migration, left. Bars represent mean  ±  SEM; (*n* = 3/experimental condition), *p*-value using Student’s *t*-test. (**C**). Schema of the co-culture of U937 and SKOV3. Bottom, Western blots showing the effect of CC on the expression of total and phosphorylated p65RelA subunit of NFκB in U937 and OvCa cocultures, with HSP90 as loading control.

We next determined the effect of CC on another key player in the OvCa TME, namely tumor associated macrophages (TAMs). The crosstalk between OvCa cells and TAMs is instigated by OvCa cells secretome attracting macrophages to the peritoneal TME and their phenotypic switch to proinflammatory tumor associated phenotype. In turn, the secretome of TAMs induces OvCa cell migration and invasion [13,59]. To elucidate the effect of CC on the OvCa cells-TAMs crosstalk, we determined the effect of CC on OvCa cells-induced macrophage chemotaxis, and macrophage-induced OvCa cell matrix invasiveness [12,13,38]. We found that CC inhibited macrophage migration towards OvCa cells, i.e., chemotaxis (Figure 8A), as well as macrophage induced OvCa cell invasiveness (Figure 8B). To further elucidate the mechanism of inhibition of U937-OvCa crosstalk, we treated mono- and co-cultures with CC for 24 h and found that CC decreased the activation and phosphorylation of p65RelA subunit of NFκB in both mono and cocultures (Figure 8C and Figure S7). Consistently, CC significantly decreased the expression of NFkB target genes in both cell types (Figure S8).

### 3.6. Compound C Suppresses Cellular Bioenergetics

**Figure 9:**
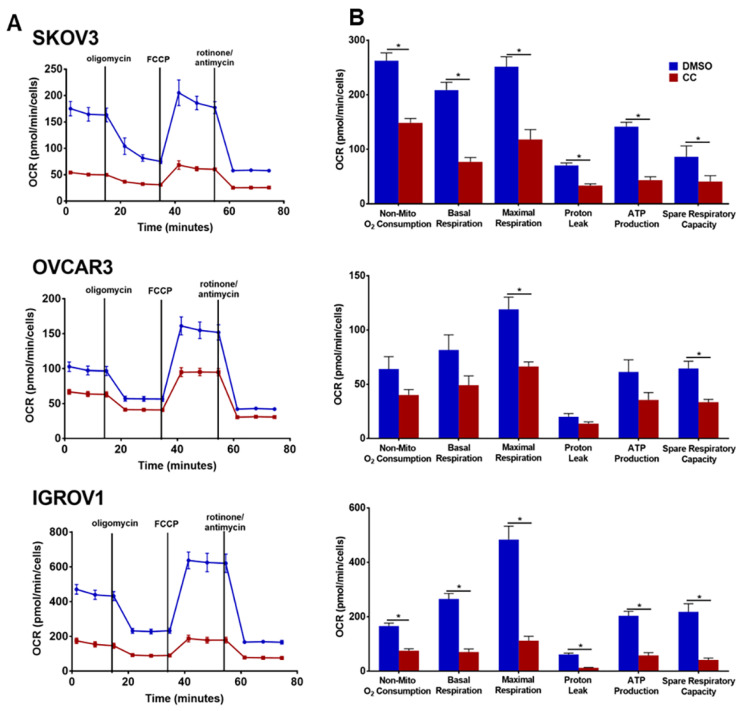
The Effect of compound C on OvCa cell bioenergetics. (**A**). Seahorse tracing of the oxygen consumption rate (OCR) in SKOV3, OVCAR3 and IGROV1 treated with CC for 18 h, followed by mitochondrial stress test as described in material and methods. (**B**). Bar graphs of means ± SEM of the basal and maximal respiration, spare respiratory capacity, ATP production, non-mitochondrial respiration, proton leak and mitoOCR/glycoPER ratio in OvCa cells treated with CC. *n* = 6/experimental condition. * p < 0.05, with multiple *t*-tests and Holm-Sidak correction.

**Figure 10:**
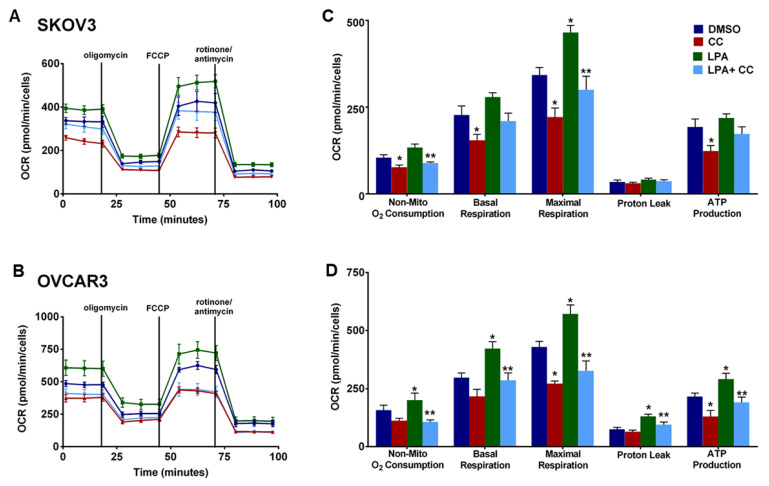
Effect of CC on LPA-Induced mitochondrial respiration. Seahorse tracing of the OCR in (**A**). SKOV3 and (**B**). OVCAR3 stimulated with LPA, in presence or absence of CC for 6 h. Bar graphs represent the means ± SEM of the basal and maximal respiration, spare respiratory capacity, ATP production, non-mitochondrial respiration, in (**C**). SKOV3 and (**D**). OVCAR3 cells treated with LPA ± CC (*n* = 5/experimental condition, repeated twice). * *p* < 0.05, compared DMSO, ** *p* < 0.05 compared to LPA, Student’s *t*-test.

Our findings of the inhibitory effect of CC on PI3K-AKT-mTOR pathway prompted us to determine whether CC also inhibits metabolic programming. Realtime monitoring of cellular bioenergetics using Seahorse mito-stress assay, revealed that CC significantly inhibited basal and maximal respiration, ATP production, and non-mitochondrial O_2_ consumption in SKOV3, OVCAR3 and IGROV1 cell lines (Figure 9A,B). We next determined whether CC mitigates the effect of LPA, the *bone a fide* activator of PI3K-AKT-mTOR pathway on cellular bioenergetics. We found that LPA treatment of OvCa cell lines SKOV3 and OVCAR3 significantly induced basal and maximal respiration, non-mitochondrial O_2_ consumption, and ATP production (Figure 10). CC significantly mitigated LPA-induced basal and maximal respiration, non-mitochondrial O_2_ consumption, and ATP production in SKOV3 and OVCAR3. These data indicate that CC exerts its inhibitory effect through inhibition of mitochondrial respiration and ATP production as measures of oxidative phosphorylation (OXPHOS). We also determined the effect of CC on glycolytic rate by measuring extracellular acidification rate (ECAR) and cellular ATP production from glycolysis. We found CC did not exert a significant effect on ECAR or ATP production from glycolysis (Figure S9A,B). We further confirmed the preferential inhibitory effect of CC on mitochondrial function as an ATP source in OvCa cells by calculating the ratio of mitochondrial respiration, represented as OCR, to that of glycolysis, represented as the proton efflux rate (PER) (Figure S10A,B). We next determined the effect of CC on mitochondrial mass using MitoTracker green-fluorescent dye [25] and found that CC did not exert significant effect on mitochondrial mass in OVCAR3 and IGROV1 cells with a trend though insignificant decrease in SKOV3 cells (Figure S10). 

### 3.7. Combination Therapy of Compound C and Cisplatin, Reduced Tumor Burden in SKOV3 Xenografts in Athymic Nude Mice

**Figure 11:**
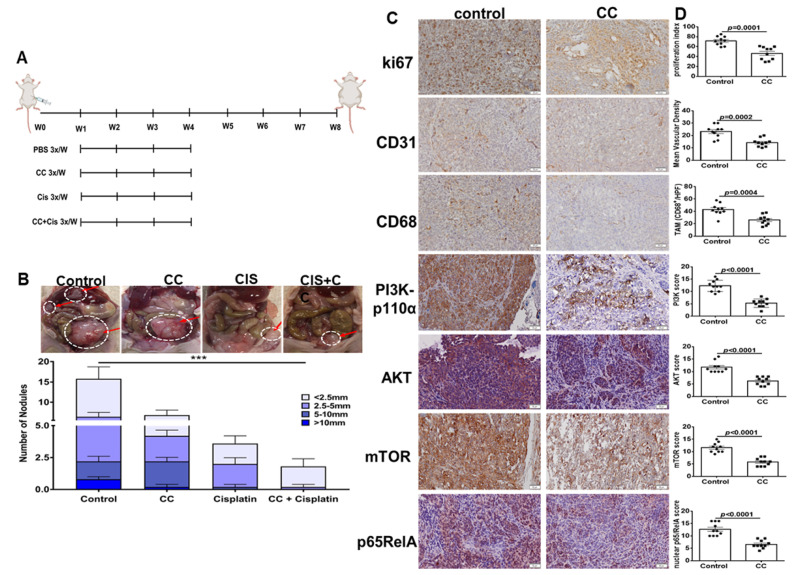
Effect of Compound C on *in vivo* tumor xenografts in athymic nude mice. (**A**). Schema of the therapeutic experiments. (**B**). Photomicrographs of intraperitoneal SKOV3 tumor nodules, top. Bars represent tumor burden (numbers and sizes) of intraperitoneal tumor nodules in the indicated experimental groups, bottom. *n* = 10/experimental condition. *** *p* < 0.001, multiple *t*-tests. (**C**). Immunostaining of representative tumor sections from mice treated with CC or PBS vehicle control (100× magnification). (**D**). Bars represent the H-scores of the indicated markers. *p*-values Student’s *t*-test.

A major challenge in OvCa treatment is chemo-resistance, specifically to platinum-derived compounds (reviewed in [60]). This prompted us to study the ability of compound C in combination with cisplatin to reduce tumor burden in vivo in OvCa cell xenografts. We injected OvCa cells intraperitoneally (ip) in athymic nude mice and ip tumor growth by IVIS bioluminescent imaging and quantification of photons flux were used for non-invasive monitoring of tumor burden in live mice. One week after tumor cell injection, mice were stratified into treatment groups that received CC, cisplatin, in mono and combinatorial therapy as well as vehicle (PBS) control (Figure 11A). Monotherapy with CC resulted in a significant decrease in tumor burden as determined the number and size of peritoneal tumor nodules (Figure 11B) by IVIS imaging and quantification of photon flux (Figure S11). Cisplatin alone significantly decreased tumor burden as determined by nodule count and size, and photon flux (Figure 11B and Figure S11). Combinatorial treatment with CC and cisplatin significantly reduced tumor burden as determined by nodule count and size and photon flux, when compared with either treatment alone (Figure 11B and Figure S11). Immunostaining of tumor excised from mice treated with CC exhibited significant decrease in proliferation index (nuclear ki67), mean vascular density (CD31), and tumor associated macrophages infiltration (CD68) immunostaining (Figure 11C,D). Furthermore, tumors from CC-treated mice exhibited significant decrease in the protein expression of PI3Kp110, pAKT, mTOR as well as nuclear p65RelA subunit of NFκB (Figure 11C,D).

### 3.8. Compound C Synergized with Cisplatin in Platinum-Resistant Patient Derived OvCa Cells

**Figure 12:**
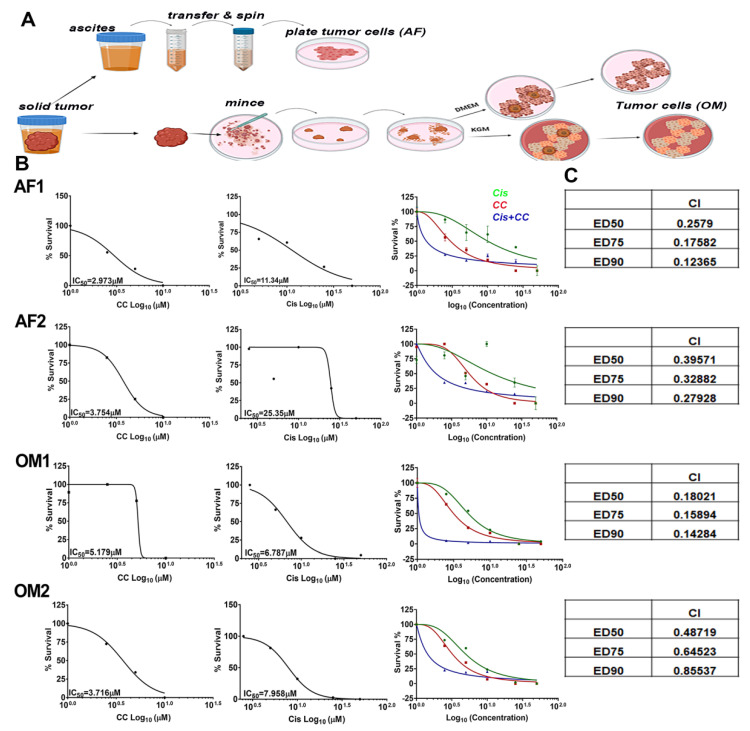
Compound C re-sensitizes cisplatin-resistant patient-derived OvCa cells to cisplatin. (**A**) Schema of isolation and maintenance of patient derived OvCa cells. (**B**) IC_50_ values of AF1, OM1, AF2 and OM2 cells, showing the effect of CC and cisplatin in mono- and combination therapy on ascitic fluid-derived (AF) and matching omentum-derived (OM) cells. (**C**) Table insets show the combination indices (CI) and the effective doses of combinatorial therapy of CC and cisplatin.

To further validate the therapeutic efficacy of CC on resistant and recurrent OvCa, we isolated OvCa cells from ascitic fluid and OvCa omental tumor of a patient with OvCa after two cycles of neoadjuvant platinum therapy (AF1, OM1: platinum-sensitive), and another patient who underwent six cycles of neoadjuvant platinum therapy (AF2, OM2: platinum resistant, Figure 12A). We found that CC significantly reduced proliferation of patient derived primary cells in a dose-dependent manner (Figure 12B). Moreover, cisplatin-sensitive AF1 was more susceptible to the cytotoxic effect of cisplatin than the cisplatin-resistant AF2 as determined by IC_50_ of 11.3 µM and 25.3 µM for AF1 and AF2, respectively; an effect that was not observed in their matching omental tumor cells OM1 and OM2 (Figure 12B). Interestingly, OM1 and OM2 were more sensitive to the cytotoxic effect of cisplatin as compared with their matching AF1 and AF2, with IC_50_ of 6.787 µM and 7.958 µM for OM1 and OM2, compared to 11.3 µM and 25.3 µM for AF1 and AF2, respectively. Interestingly, treatment of the four cell types with their respective IC_50_ concentration of CC and cisplatin exerted a synergistic effect in all four cell populations with combination indices < 1 (Figure 12B,C), strongly suggesting the potential efficacy of CC in conjunction with current chemotherapy to overcome resistance in high-grade ovarian tumors.

## 4. Discussion

Our current study demonstrates the potent inhibitory effects of CC in OvCa. First, we show that CC inhibits malignant phenotypes of OvCa including cell survival, proliferation, migration, and matrix invasion. We further showed that CC inhibited OvCa-stromal interactions, namely with mesothelial cells and macrophages, and demonstrated the inhibitory effect of CC on the expression and activation of the most amplified and hyperactivated oncogenic pathways in OvCa patients namely, PI3K-AKT-mTOR-NFκB. The inhibitory effect of CC on OvCa cells interactions with mesothelial cells and macrophages is mediated through inhibition of the expression and activation of p65RelA subunit of NFκB in both cell types. Importantly, CC inhibited mitochondrial bioenergetics, with modest effect on mitochondrial mass in OvCa cells. In vivo, CC in combination with cisplatin, decreased tumor burden, and corroborated our in vitro studies demonstrating its strong synergy with cisplatin in both platinum sensitive and resistant patient derived OvCa ascitic fluid and solid omental tumors. 

The anti-tumorigenic effects of CC have been studied in several cancers including colon, ovarian, and breast cancers, glioma and B-cell lymphoblastic leukemia [32,61,62,63,64], and have historically been attributed to inhibition of AMPK and BMP pathways [64]. However, AMPK- and BMP- independent tumoricidal activity were reported [29,64,65]. Comparing the anti-tumor activity of CC to those of AMPK putative activators, AICAR and the potent biguanide phenformin, we found that CC exerted a more potent tumoricidal effect on OvCa cell lines in micromolar concentrations compared to AICAR and phenformin that exerted their effects in millimolar concentrations. In addition, re-analysis of published transcriptomic data of OvCa cell line SKOV3 treated with CC using GSEA, identified inhibitory effect of CC on PI3K-AKT-mTOR, the most commonly hyperactivated pathway with activating mutations, and amplification in many cancers including OvCa [7,8]. The anti-tumorigenic effect of CC has been also attributed to its inhibitory effect on angiogenesis through an effect on endothelial cells in in vitro assays and inhibition of tumor angiogenesis in a B16 melanoma mouse model [33,34]. This anti-angiogenic effect of CC was further supported by inhibition of the angiogenesis signature in SKOV3 treated with CC *GSE60135* [40] (Figure S11C). Our data in the present study further highlighted the anti-angiogenic effect of CC not only through a direct effect on OvCa cells but through an effect on the interactions of OvCa cells with macrophages and mesothelial cells in co-cultures that significantly induced VEGF transcripts in these cells. The reciprocal feedback loop between angiogenesis and PI3K-AKT-mTOR and NFκB pathways has long been established [66,67,68,69,70,71]. Thus, our findings herein that CC inhibits VEGF transcript expression in vitro as well as angiogenesis of OvCa cell xenografts in vivo further highlight the therapeutic utility of CC in OvCa.

The role of CC as an activator or inhibitor of PI3K-AKT-mTOR-NFκB pathways is contextual not only disease-specific but are also model system-specific with substantial technical variabilities in the studies reporting such role [65,72,73,74]. Furthermore, known chemical modifications and/or homologs of CC were designed to enhance its effects on BMP/BMP receptors as an anti-fibrotic agent in pulmonary fibrosis and diabetic nephropathy [75,76,77]. However, the therapeutic efficacy of these molecules on OvCa cells was understudied with only two studies reporting the therapeutic potential of CC in OvCa [32,40]. Our report in the present study highlights a novel compelling and multi-faceted anti-tumorigenic effect of CC that warrants further development of CC for the treatment of OvCa as an inhibitor of PI3K-AKT-mTOR-NFkB axis. Importantly, we showed that the significant suppression of proliferation, angiogenesis, TAM infiltration as well as the expression and activity of PI3K and nuclear p65RelA in SKOV3 xenografts from mice treated with CC. Moreover, we also found that CC exerted a significant cytotoxic effect on platinum- sensitive and platinum-resistant omental and ascitic fluid patient-derived primary OvCa cells (OM1, AF1, OM2, and AF2, respectively). Hence, our findings suggest that CC works synergistically with cisplatin to inhibit OvCa progression in vivo and in vitro. In the literature, only one earlier study demonstrated that CC increased animal survival of A2780-s xenografts; however, this study focused on the inhibitory effect on CC on BMP-SMAD5 pathway [32]. 

The complex OvCa milieu is dictated by the bioenergetic alterations of the OvCa cells themselves and thus we sought to determine the effect of CC on OvCa cells bioenergetics. We found that CC inhibited oxidative phosphorylation, in particular suppressing basal and maximal respiration, spare respiratory capacity, ATP production, non-mitochondrial respiration, and proton leak in three OvCa cell lines, while having modest though insignificant effects on the glycolysis and fatty acid oxidation. Importantly, CC mitigated the stimulatory effect of LPA, initially reported as OvCa promoting factor and a putative activator of PI3K-AKT-mTOR and NFκB [78,79] on mitochondrial bioenergetics and ATP production. Given our earlier reports of the reliance of OvCa stem cells on oxidative phosphorylation [80], our results further suggest that CC could be used to target OvCa stem cells and prevent relapse. 

Several clinical trials are currently underway that target PI3K pathway either as single agents (NCT01068483, NCT01833169, NCT04836663, NCT02307240, NCT04586335, NCT01936363, NCT01708161), or in combination with standard of care platinum, taxane compounds and the more recent poly-ADP ribose polymerase inhibitors, PARPi (NCT05295589, NCT00216112, NCT03586661). However, as earlier summarized [55,56,81], these agents did not go beyond phase 1 or early phase 2 clinical trials with reported adverse effects as hyperglycemia, myopathies, mucositis, and neuropathies that halted progression to advanced phases of trials [81]. Our study shows that while CC exhibited a significant anti-tumor effect in vitro, its effect as a single agent was not as potent as cisplatin. This is also consistent with the use of PI3K inhibitors in clinical trials in combination with standard of care therapeutics. Our finding that CC not only exerted a synergistic effect with cisplatin in vitro and in vivo, but it also sensitized cisplatin resistant OvCa cells to cisplatin, would justify its use in combination with cisplatin to improve the disease outcome, reduce the dose of cisplatin and the adverse events of cisplatin therapy.

## 5. Conclusions

The challenge in developing novel treatment options for OvCa lies in the fact that there is significant upregulation of oncogenic feedback mechanisms, and only a few of the current therapeutics target the interactions of OvCa in the TME that drive recurrence and resistance. Thus, our findings that CC inhibits the interactions of CC with mesothelial cells and macrophages further highlight CC as a promising therapeutic agent especially given that it was well tolerated in the in vivo preclinical models. Our findings that computational modeling showed docking of CC into PI3K, mTOR and p65RelA warrant further development of CC (and perhaps analogs) to improve potency as a multi-valent therapeutic. 

## Data Availability

The data presented in this study are available in the article and the Supplementary Materials.

## Supplementary Materials

The following supporting information can be downloaded at: https://www.mdpi.com/article/10.3390/cancers14205099/s1, Figure S1: Effects of phenformin and AICAR on colony formation of OvCa cell lines. A–C. SKOV3, OVCAR3, and IGROV1 were treated with AICAR (0–2 mM), and D–E. phenformin (0–1 mM). All experiments were performed in triplicates/experimental condition and were repeated at least twice; **** *p* < 0.0001, one way Analysis of Variance (ANOVA); Figure S2: Full WBs of Figure 4C; Figure S3: Full WBs of Figure 4D; Figure S4: Full WBs of Figure 6A; Figure S5: Full WBs of Figure 7D; Figure S6: Effect of CC on the expression of NFkB target genes in SKOV3 and mesothelial cells in mono- and co-cultures. Bras represent the means ± SEM of the relative expression of the transcripts of the indicated genes determined by qRT-PCR. * *p* < 0.05, comparing CC- to DMSO-treated cells. ** *p* < 0.05, comparing cells in mono- to cocultures, multiple *t*-tests; Figure S7: Full WBs of Figure 8C; Figure S8: Effect of CC on the expression of NFkB target genes in SKOV3 and U937 macrophages in mono- and co-cultures. Bras represent the means ± SEM of the relative expression of the transcripts of the indicated genes determined by qRT-PCR. * *p* < 0.05, comparing CC- to DMSO-treated cells. ** *p* < 0.05, comparing cells in mono- to cocultures, multiple *t*-tests; Figure S9: The Effect of CC on glycolytic rate. A. Seahorse tracing of the proton efflux rate (PER) in SKOV3 and OVCAR3 treated with 5 µM of CC for 18 h, as described in material and methods. B. Bars representing means ± SEM of the basal and compensatory glycolysis, post 2DG acidification, basal proton efflux rate,%PER from glycolysis and mitochondrial oxygen consumption rate/glycolysis proton efflux rate (mitoOCR/glycoPER) ratio in SKOV3 and OVCAR3 cells treated with CC. A–B are representatives of 3 experiments (*n* = 3/experimental condition) * *p* < 0.05, Student’s *t*-test; Figure S10: The effect of CC on mitochondrial mass. A. Confocal microscopy images of MitoTracker and merged images of SKOV3, OVCAR3 and IGROV1 cells treated with CC or DMSO control for 18 h (scale bar, 10 µm) B. Bar graphs represent means ± SEM of quantifies fluorescent pixel intensity of using ImageJ. *p*-values are determined by Student’s *t*-test, comparing CC- to DMSO-treated groups; Figure S11: (A) IVIS imaging of tumor-bearing mice 8-weeks after ip injection of SKOV3-luc, and (B) box plots of the quantification of the photon flux/second in the experimental in the experimental cohorts. * *p* < 0.05, compared to the vehicle control. # *p* < 0.05 compared to monotherapy with either cisplatin (cis) and CC, Mann–Whitney’s test. (C) GSEA analysis of SKOV3 cells treated with CC showing enrichment of a signature of angiogenesis; Table S1: List of Antibodies Used; Table S2: List of primers used.
