# Supplementary material for: Compound C Inhibits Ovarian Cancer Progression via PI3K-AKT-mTOR-NFκB Pathway
Source: Cancers (Basel). 2022 Oct 18;14(20):5099. doi: 10.3390/cancers14205099 (PMC9600774; doi:10.3390/cancers14205099)
Supplement: Supplementary file 1 [file cancers-14-05099-s001.zip › cancers-1934725-supplementary.pdf]

Table S1. List of Antibodies Used

Primary and secondary Antibodies	Catalog #	Dilution	Molecular weight	Application
Rabbit mTOR (7C10) (Cell Signaling Technology CST, Danvers, MA)	2983	1:1000<br>1:50	289kDa	WB<br>IHC
Rabbit Phospho-mTOR (Ser2448) (D9C2) (CST)	5536	1:500	189kDa	WB
Rabbit Anti-p70 S6 Kinase (S6K) (Sigma)	06-926	1:1000	70kDa	WB
Rabbit Phospho-p70 S6 Kinase (Thr389) (108D2) (CST)	9234	1:500	70kDa	WB
Rabbit AKT (CST)	9272	1:1000	60kDa	WB
Rabbit Phospho-AKT (Ser473) (CST)	4060	1:500<br>1:100	60kDa	WB<br>IHC
Rabbit HSP90 (CST)	4874	1:1000	90kDa	WB
Rabbit NFκB p65 (D14E12) XP (CST)	8242	1:1000<br>1:200	65kDa	WB<br>IHC
Rabbit Phospho-NFκB p65 (Ser536) (93H1) (CST)	3033	1:500	65kDa	WB
Rabbit PI3 Kinase p85 (19H8) (CST)	4257	1:1000	85kDa	WB
Rabbit PI3 Kinase 110α (C73F8) (CST)	4249	1:500	110kDa	WB
Rabbit polyclonal PI3 Kinase p110α (Proteintech Group, Inc., Rosemont, IL)	20583-1-AP	1:50	110kDa	IHC
Rabbit IgG HRP Linked Whole Ab (Novus Biologicals, LLC, Centennial, CO)	NA934	1:1000	--	WB
Rabbit IKKα (D3W6N) (CST)	61294	1:500	85kDa	WB
β-Actin loading control monoclonal antibody (BA3R; Invitrogen)	MA5-15739	1:5000	42kDa	WB
Rabbit CD31 (NovusBio)	NB100-2284	1:50	82.5 kDa	IHC
Rabbit Ki67 (NovusBio)	NB500-170	1:100	319 kDa	IHC
Mouse RelA/p65 (CST)	6956s	1:100	65 kDa	IHC
Mouse CD68 (NovusBio)	NB100-683	1:200	110kDa	IHC
Rabbit Alexa Flour 594 Conjugate (Invitrogen)	8889	1:500	--	IF
Rabbit Alexa Flour 488 Conjugate (Invitrogen)	7395	1:400	--	IF

Table S2: List of primers used:

Gene	Forward Primer sequence [5'-3'] Oligo	Reverse Primer sequence [5'-3'] Oligo
IL6	TACCCCCAGGAGAAGATTCC	GCCATCTTTGGAAGGTTCAG
CCL2	CCCCAGTCACCTGCTGTTAT	GAGTTTGGGTTTGCTTGTCC
IL8	GTGCAGTTTTGCCAAGGAGT	CTCTGCACCCAGTTTTCCTT
TNFα	AACCTCCTCTCTGCCATCAA	CCAAAGTAGACCTGCCCAGA
VEGF	CCTTGCTGCTCTACCTCCAC	CACACAGGATGGCTTGAAGA
IL1b	CAGAAGTACCTGAGCTCGCC	AGATTCGTAGCTGGATGCCG
18S	GCGGTTCTATTTTGTTGGTTT	CTCCGACTTTCGTTCTTGATT

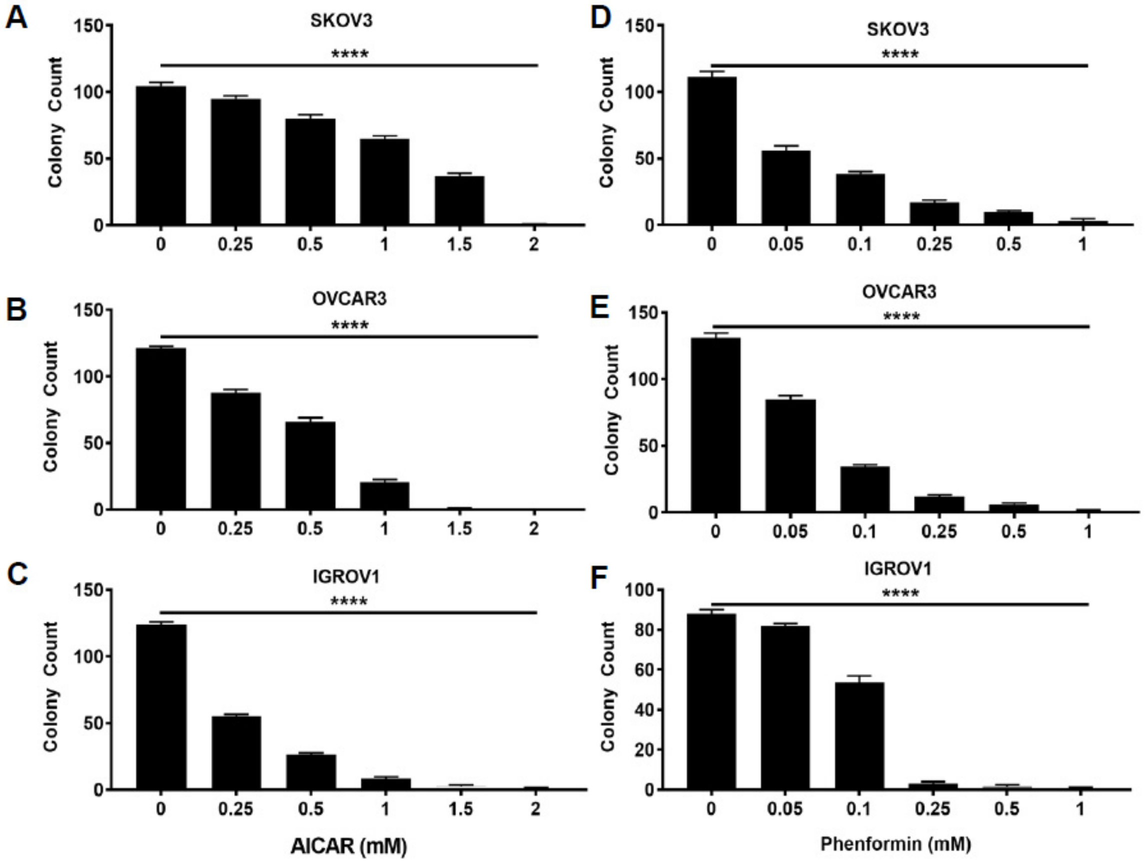

Figure S1. Effects of phenformin and AICAR on colony formation of OvCa cell lines. A–C. SKOV3, OVCAR3, and IGROV1 were treated with AICAR (0-2mM), and **D–E.** phenformin (0-1mM). All experiments were performed in triplicates/experimental condition and were repeated at least twice; ****p< 0.0001, one way ANOVA).

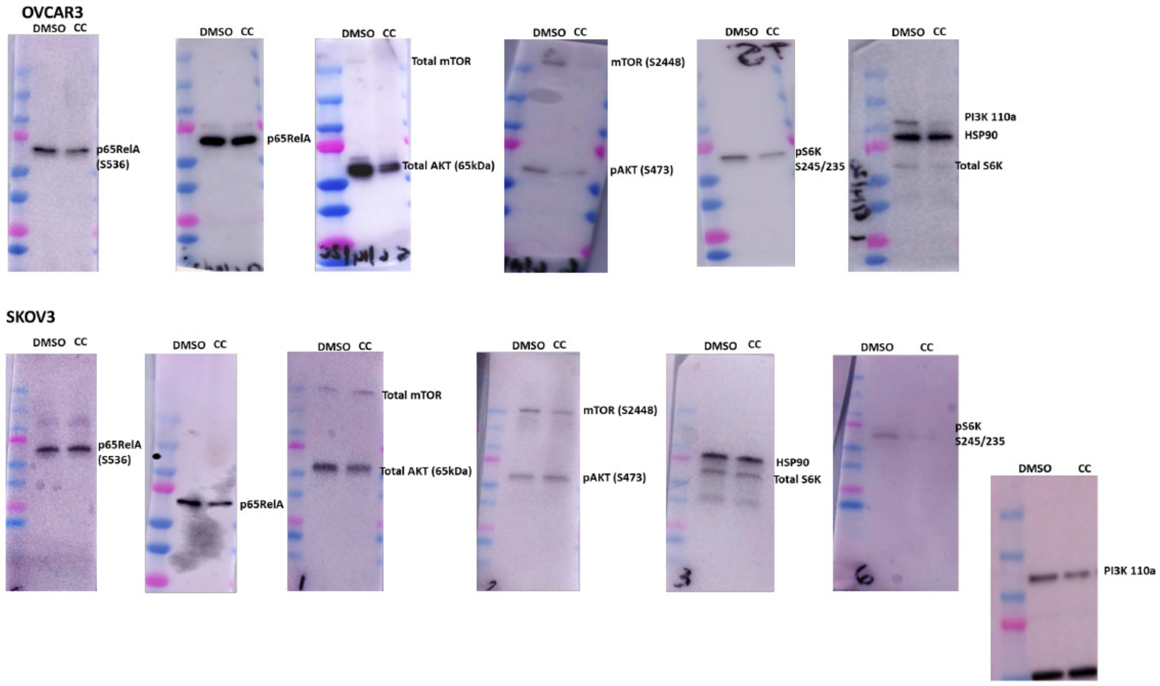

Figure S2. Full WBs of Figure 4C.

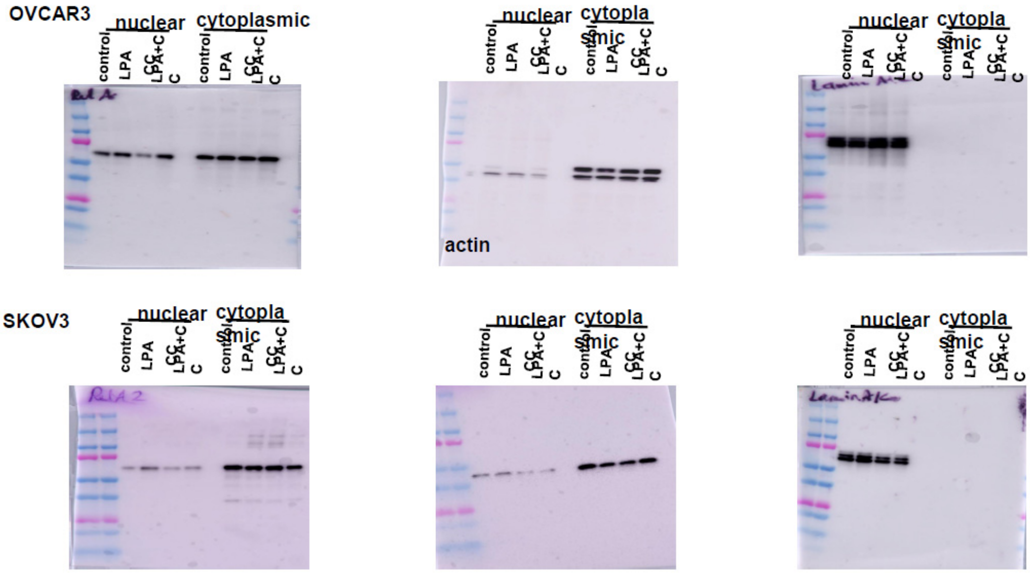

Figure S3. Full WBs of Figure 4D.

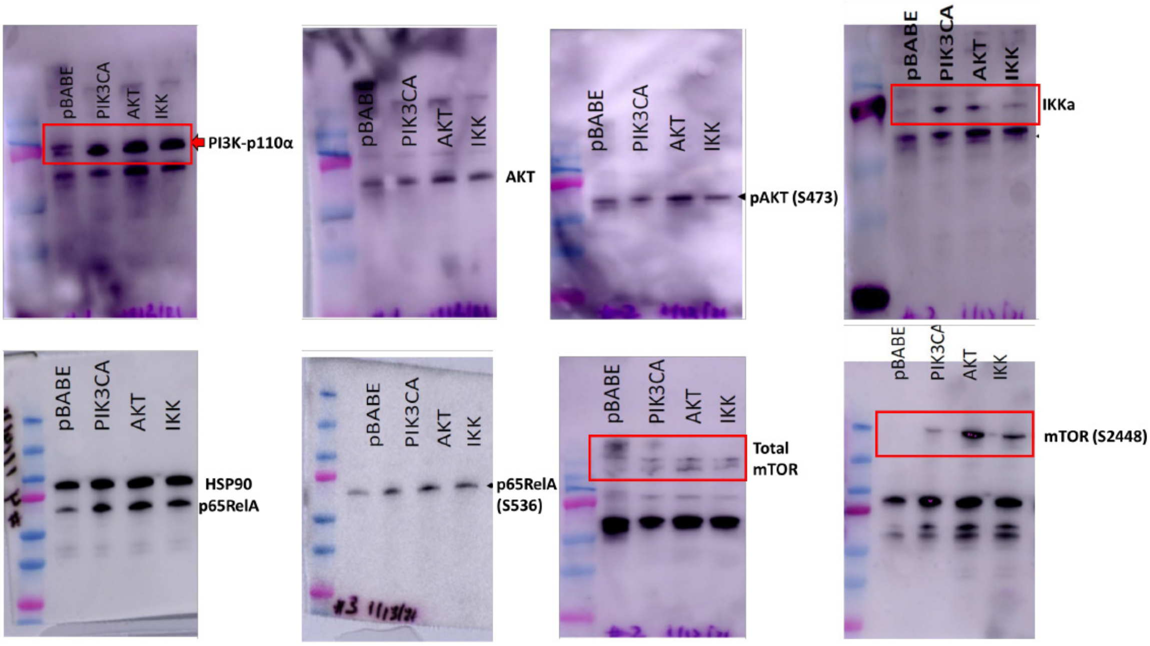

Figure S4. Full WBs of Figure 6A.

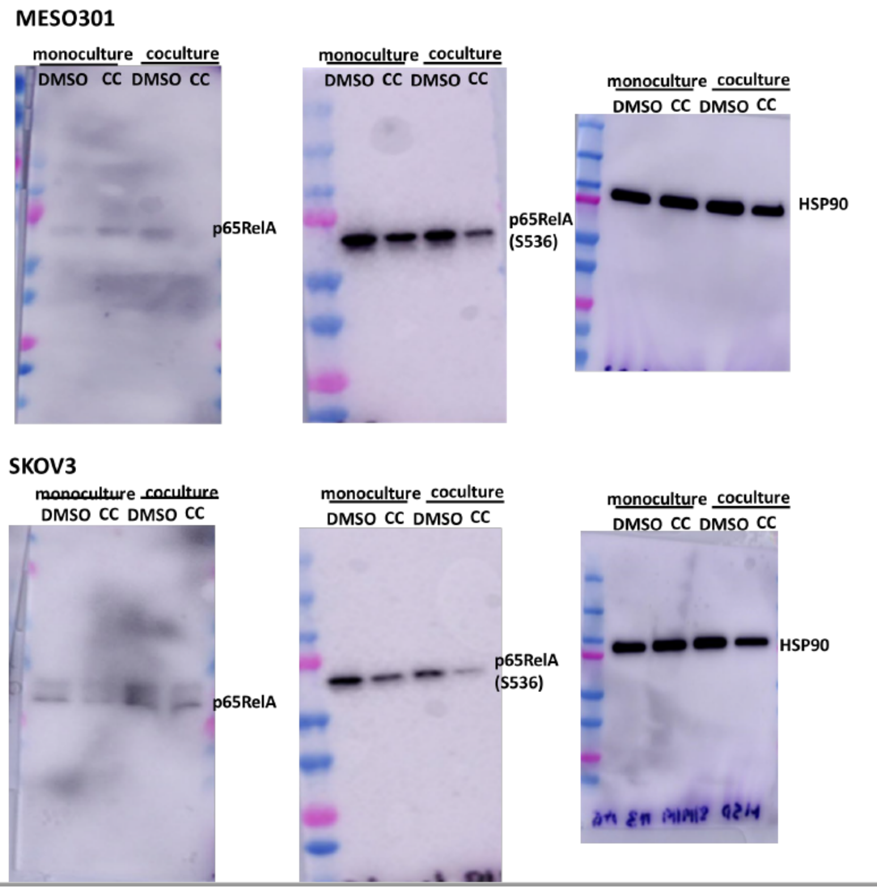

Figure S5. Full WBs of Figure 7D.

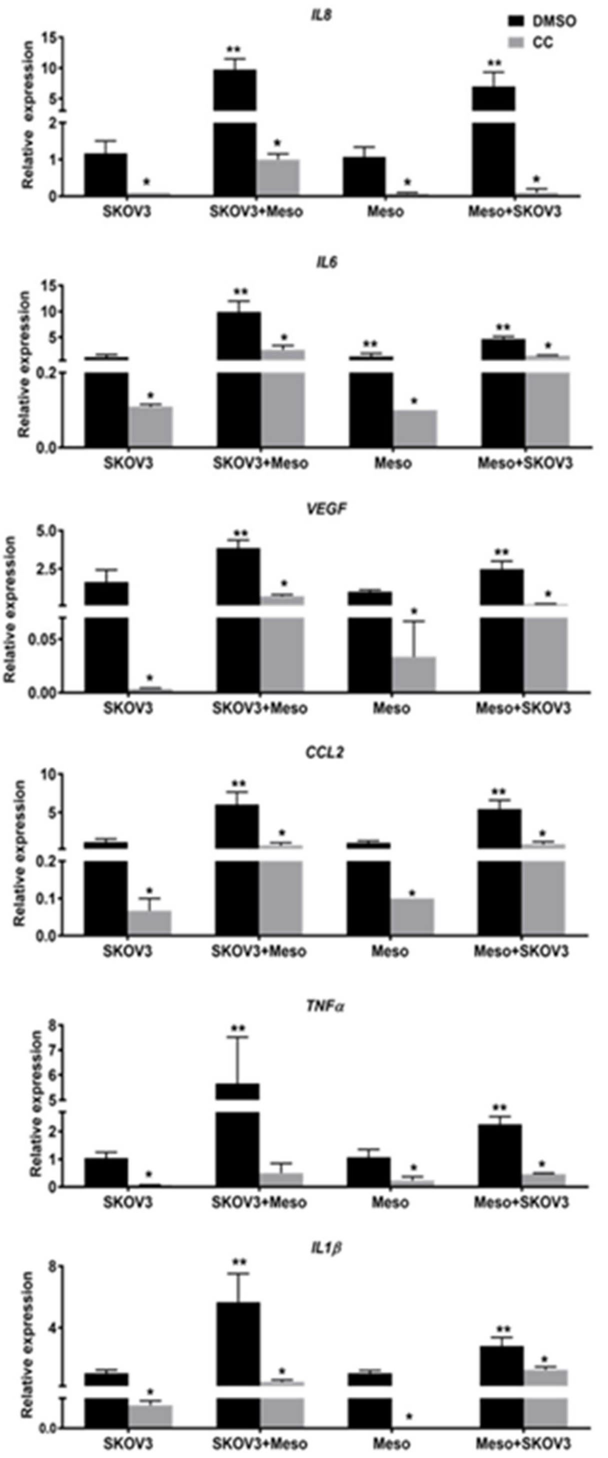

Figure S6. Effect of CC on the expression of NFkB target genes in SKOV3 and mesothelial cells in mono- and co-cultures. Bras represent the means ± SEM of the relative expression of the transcripts of the indicated genes determined by qRT-PCR. **p*<0.05, comparing CC- to DMSO-treated cells. ***p*<0.05, comparing cells in mono- to cocultures, multiple *t*-tests.

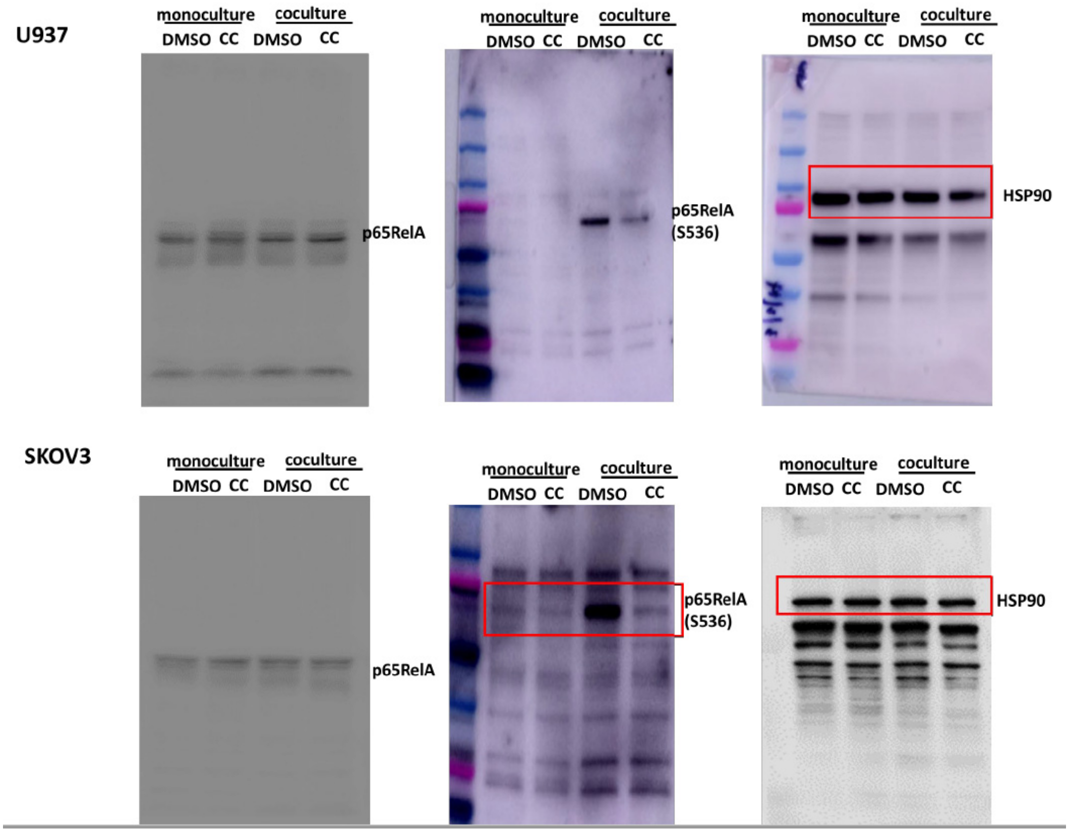

Figure S7. Full WBs of Figure 8C.

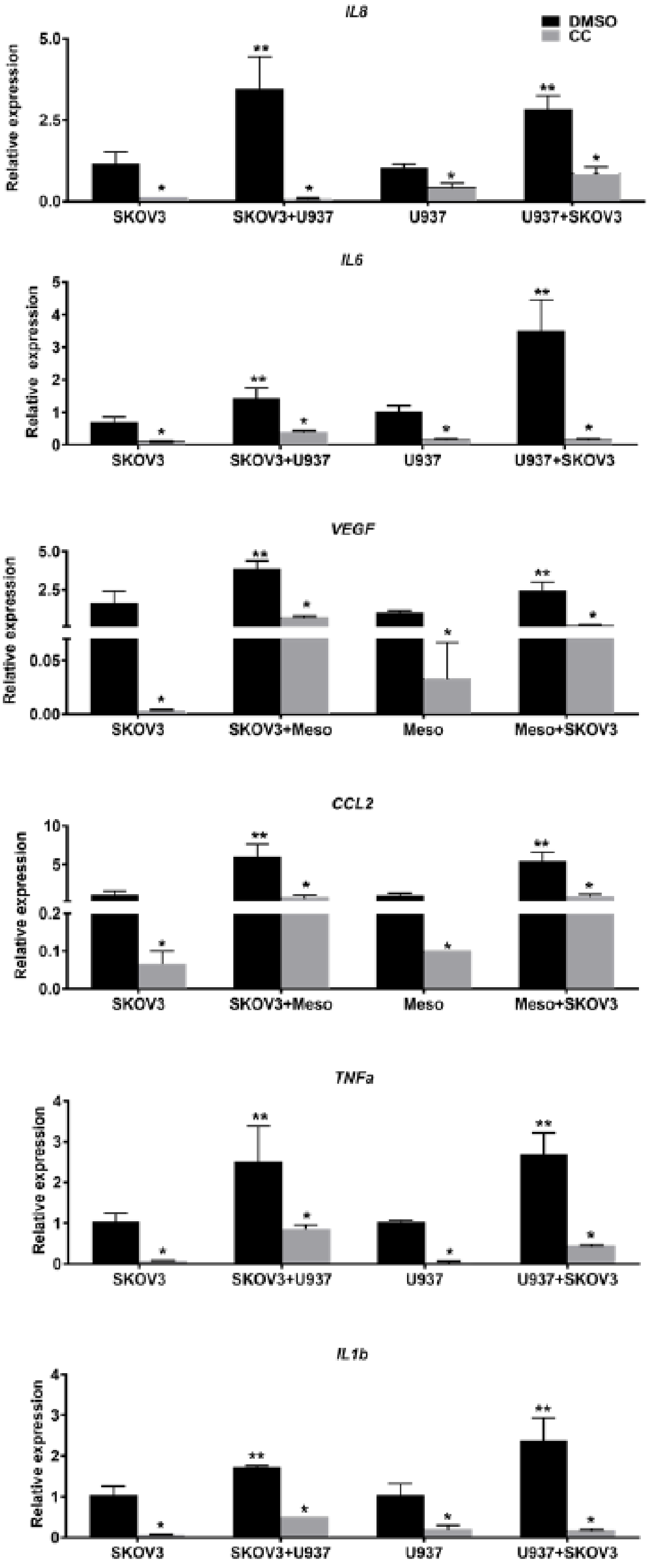

Figure S8. Effect of CC on the expression of NFkB target genes in SKOV3 and U937 macrophages in mono- and co-cultures. Bras represent the means ± SEM of the relative expression of the transcripts of the indicated genes determined by qRT-PCR. *p<0.05, comparing CC- to DMSO-treated cells. **p<0.05, comparing cells in mono- to cocultures, multiple t-tests.

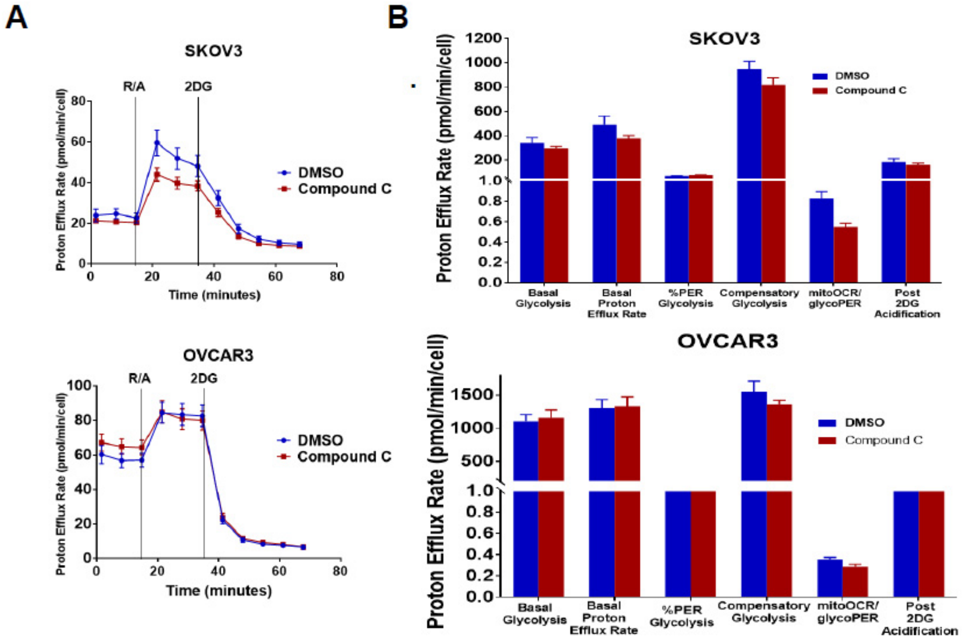

Figure S9. The Effect of CC on glycolytic rate. A. Seahorse tracing of the proton efflux rate in SKOV3 and OVCAR3 treated with 5μM of CC for 18hr, as described in material and methods. **B.** Bars representing means ± SEM of the basal and compensatory glycolysis, post 2DG acidification, basal proton efflux rate, %PER from glycolysis and mitoOCR/glycoPER ratio in SKOV3 and OVCAR3 cells treated with CC. **A-B** are representatives of 3 experiments (n=3/experimental condition) **p*< 0.05, Student's *t*-test.

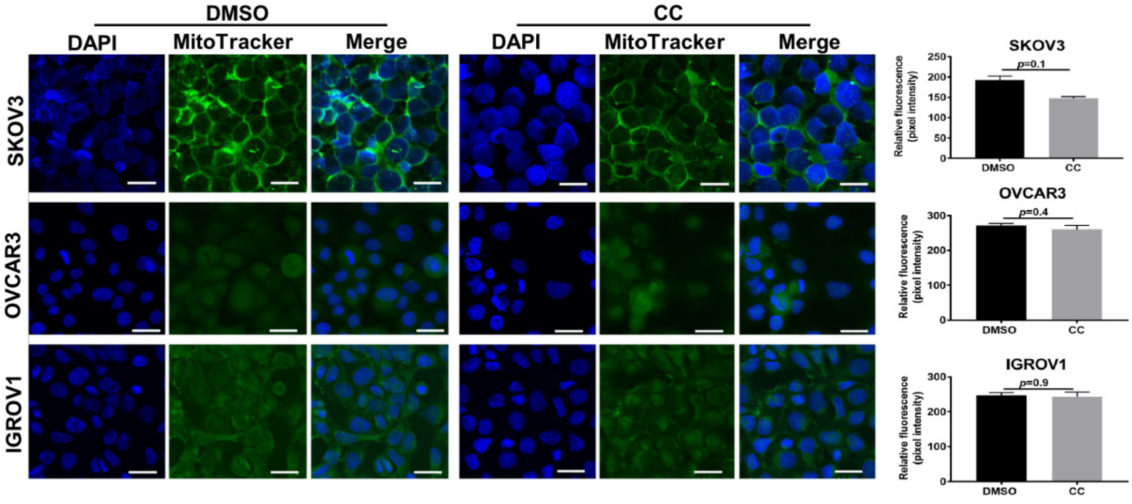

Figure S10. The effect of CC on mitochondrial mass. A. Confocal microscopy images of Mito-Tracker and merged images of SKOV3, OVCAR3 and IGROV1 cells treated with CC or DMSO control for 18hrs (scale bar, 10μm) **B.** Bar graphs represent means ±SEM of quantifies fluorescent pixel intensity of using Image J. *p*-values are determined by Student's *t*-test, comparing CC- to DMSO-treated groups.

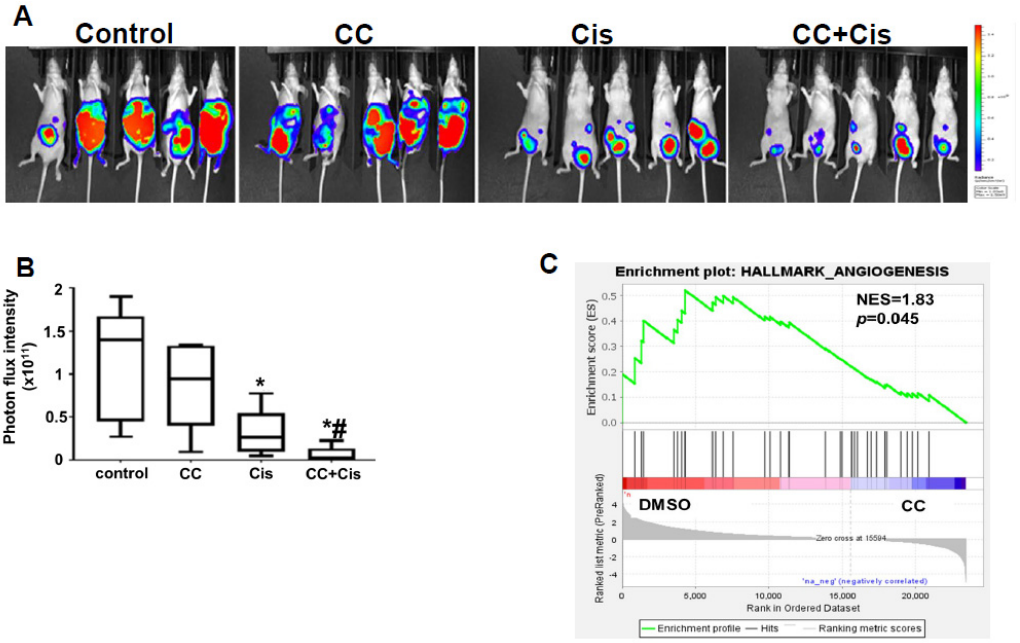

Figure S11. A. IVIS imaging of tumor-bearing mice 8-weeks after ip injection of SKOV3-luc, and **B.** box plots of the quantification of the photon flux/second in the experimental in the experimental cohorts. **p*<0.05, compared to the vehicle control. #*p*<0.05 compared to monotherapy with either cisplatin (cis) and CC, Mann-Whitney's test. C. GSEA analysis of SKOV3 cells treated with CC showing enrichment of a signature of angiogenesis.
